# Editorial: Unveiling the complexity of neuroendocrine neoplasms: biomarkers, classification, and translational perspectives

**DOI:** 10.3389/fendo.2026.2030369

**Published:** 2026-10-28

**Authors:** Anna La Salvia, Giuseppe Fanciulli

**Affiliations:** 1 National Center for Drug Research and Evaluation, National Institute of Health (ISS), Rome, Italy; 2 Neuroendocrine Tumor Unit, Department of Medicine, Surgery and Pharmacy, University of Sassari, Sassari, Italy; 3 Endocrine Unit, Azienda Ospedaliero-Universitaria (AOU) of Sassari, Sassari, Italy

**Keywords:** biomarker, classification, molecular and epigenetic profiling, neuroendocrine neoplasms (NENs), real world evidence (RWE), translational perspectives

Neuroendocrine neoplasms (NENs) encompass a heterogeneous family of tumors that share neuroendocrine differentiation but differ substantially in anatomical origin, molecular background, clinical behavior, functional status, and prognosis. This diversity has long represented one of the major challenges in the field. However, increasing biological, pathological, and clinical evidence suggests that such complexity should not be regarded solely as a source of diagnostic uncertainty. Rather, it may provide the biological substrate from which more refined classification systems, prognostic assessment, and translational investigation can emerge.

The Research Topic *Unveiling the Complexity of Neuroendocrine Neoplasms: Biomarkers, Classification, and Translational Perspectives* was conceived to explore this evolving scenario. Its aim was to bring together studies addressing the diagnostic, biological, and translational dimensions of NENs, with particular attention to biomarkers, molecular profiling, classification, risk stratification, and real-world evidence (RWE). Taken together, the articles included in this collection provide a broad perspective on the changing landscape of NEN research, supporting a conceptual shift from static disease descriptors toward integrated frameworks.

One of the central themes emerging from this Research Topic is the refinement of classification through tissue-based biomarkers and molecular taxonomy. In pancreatic neuroendocrine tumors (PanNETs), the integration of markers such as ATRX, DAXX, PDX1, ARX, and somatostatin receptors offers a more nuanced view of tumor biology, linking lineage-related features, chromatin-associated alterations, receptor expression, and clinicopathological behavior (Ciobanu et al.). This approach shifts classification from morphology alone toward biologically informed systems that identify distinct tumor subsets within apparently homogeneous entities.

A similar principle applies to the exploration of non-conventional and transversal biomarkers. Alpha-fetoprotein, although traditionally associated with other malignancies, may provide additional biological and prognostic information in selected gastroenteropancreatic NENs (GEP-NENs), particularly when interpreted in relation to tumor grade, proliferative activity, and disease extent (La Salvia and Fanciulli). In parallel, Stathmin 1 represents an example of a potential cross-entity biomarker across NENs, with biological, pathological, and translational implications (La Salvia et al.). Thereby, biomarker discovery in NENs should extend beyond canonical neuroendocrine markers to include molecules associated with proliferation, lineage identity, progression, and aggressive behavior.

The interpretation of neuroendocrine markers themselves also requires caution. The biological and prognostic meaning of neuroendocrine marker expression in colorectal carcinoma illustrates the limits of a purely immunophenotypic approach (Mangogna et al.). Marker expression does not necessarily equate to true neuroendocrine identity, nor does it always define a uniform biological category. This has broader implications for the NEN field: diagnostic precision depends not only on detecting markers, but also on understanding their context, distribution, intensity, association with morphology, and relationship with clinical behavior. In this sense, biomarker-based classification must remain biologically grounded rather than merely descriptive.

Molecular and epigenetic profiling further expands this integrated framework. The investigation of methylation programs associated with chromosome 18 loss in small intestinal NETs (SI-NETs) links recurrent genomic alterations to broader epigenetic states (Axling et al.). Moving beyond single-marker approaches, integrating genomic, epigenomic, transcriptomic, and pathological data may better capture tumor evolution, biological subgroups, and prognostic heterogeneity, defining clinically relevant molecular classes of NENs.

A second major theme is the shift toward clinically meaningful risk stratification. Although grade, stage, differentiation, and anatomical site remain essential prognostic factors in NENs, they do not fully capture disease complexity. The NUTRIGETNE study expands risk assessment in GEP-NENs by predicting malnutrition and sarcopenia (Argente-Pla et al.), highlighting the relevance of nutritional status, muscle mass, and functional vulnerability for outcomes, treatment tolerance, quality of life, and long-term management.

The same need for refined risk assessment emerges in functioning PanNETs such as insulinomas. The early prediction of malignancy using tumor size and biochemical markers addresses a clinically relevant problem: although most insulinomas behave indolently, the timely identification of aggressive potential remains challenging (Mathew et al.). Combining anatomical and biochemical information may help distinguish patients who require closer surveillance and more individualized diagnostic pathways. Similarly, the recognition of bone metastases as a high-risk feature in metastatic NETs, particularly in NETs of unknown primary origin, highlights the prognostic relevance of disease distribution (Casabella et al.). These studies suggest that risk stratification should increasingly integrate tumor biology, functional phenotype, disease burden, metastatic pattern, and host-related factors.

The Research Topic highlights the biological and clinical determinants of NET development within specific tissue contexts. Type 1 gastric NETs associated with autoimmune atrophic gastritis exemplify the interplay between chronic inflammation, endocrine cell alterations, and the gastric microenvironment (Rossi et al.). Integrating pathological context, host-related factors, and tumor biology may support individualized surveillance strategies and advance translational research.

RWE is essential in rare tumors. The Italian Association for Neuroendocrine Tumors survey on pheochromocytomas and paragangliomas (PPGLs) reveals variability in diagnostic and clinical practice, highlighting the need for harmonization across centers (Pietroluongo et al.). RWE identifies gaps between guidelines, expert consensus, and daily practice, helping optimize biochemical testing, imaging, genetic assessment, multidisciplinary evaluation, and follow-up by assessing the implementation of classification and diagnostic principles in clinical settings.

Collectively, this Research Topic highlights four converging directions in NEN research: biomarker-informed classification incorporating tissue markers, receptor profiles, lineage factors, and non-conventional molecules (Ciobanu et al., La Salvia and Fanciulli, La Salvia et al.); molecular and epigenetic profiling to refine biological taxonomies (Mangogna et al., Axling et al.); prognostic stratification integrating grade, stage, nutritional status, biochemical phenotype, metastatic patterns, and disease-specific risk factors (Argente-Pla et al., Mathew et al., Casabella et al.); and translational models integrating pathology, molecular data, clinical phenotype, and RWE into reproducible, clinically applicable frameworks (Rossi et al., Pietroluongo et al.).

NEN complexity should be viewed as an opportunity for precision classification and individualized risk assessment. Future research should validate emerging biomarkers in large, well-annotated cohorts, integrate multi-omic data with histopathology, imaging, and clinical outcomes, develop robust predictive models, and harmonize diagnostic criteria across heterogeneous entities, supported by sustained multidisciplinary collaboration and patient-centered research networks.

In conclusion, this Research Topic provides a timely synthesis of the evolving diagnostic, biological, and translational paradigms in NENs. The articles gathered here show that the future of NEN research lies in moving from fragmented descriptors toward integrated models of disease identity and risk. By combining morphology, biomarkers, molecular and epigenetic profiling, functional status, clinical phenotype, and RWE, the field can advance toward more precise classification, earlier recognition of aggressive behavior, and better-informed translational research.

We sincerely thank all authors, reviewers, and the Frontiers editorial team for their valuable contributions to this Research Topic.

